# Obesity reprograms the normal pancreas and pancreatic cancer microbiome in mice and humans

**DOI:** 10.3389/frmbi.2025.1543144

**Published:** 2025-07-28

**Authors:** Raquel Santana da Cruz, Shravanthy Suguru, Sara P. C. Paiva, Ijeoma Nwugwo, Bhaskar Kallakury, Benjamin A. Weinberg, Katherine L. Cook, Sonia de Assis

**Affiliations:** ^1^ Department of Oncology, Lombardi Comprehensive Cancer Center, Georgetown University, Washington, DC, United States; ^2^ Department of Cancer Biology, Wake Forest University School of Medicine, Winston-Salem, NC, United States

**Keywords:** PDAC, pancreatic ductal adenocarcinoma, obesity, microbiome, mouse, human

## Abstract

**Introduction:**

Pancreatic ductal adenocarcinoma (PDAC) is an aggressive form of pancreatic cancer, with overall 5-year survival rates of about 8%. Obesity (and underlying metabolic dysfunction) is estimated to account for up to 50% of all PDACs. Microbial communities can be modulated by obesity and exert biological effects on tissues they colonize as well as distant sites. Recent studies showed that tumors, including PDAC, harbor a microbiome that is able to regulate cancer outcomes such as tumor progression, response to therapy and overall survival. Yet, it is not understood whether patient’s characteristics impact this relationship.

**Methods:**

We examined the influence of obesity (defined by body weight in mice or body mass index [BMI] in humans) on the normal and cancerous pancreas microbiome in mice and humans using 16S sequencing.

**Results:**

Overall, we observed that diet-induced obesity accelerated PDAC progression in the KC mouse model of PDAC. We also detected an obesity-induced decrease in the microbial abundance of the normal or cancerous pancreas. Obesity modified the bacterial community composition in the normal pancreas and PDAC of both mice and humans. Further, obese animals and humans each had a distinctive pancreatic microbiome signature with specific bacterial phylum, genus and species compared to controls. Notably, both the normal mouse pancreas and human PDAC showed an obesity-induced decrease in Pseudomonadota phylum. We also found that the presence of cancer by itself reduced microbial diversity in both the pancreas as well the intestinal microbiota. This reduction in microbial richness was further exacerbated by obesity. Finally, we observed that obesity increased inflammatory cytokines and altered the tumor immune infiltrate in humans and mice.

**Discussion:**

Further investigation of obesity-driven microbial differences in the pancreas could provide important insights for personalized treatments for PDAC patients.

## Introduction

Pancreatic ductal adenocarcinoma PDAC is a highly aggressive form of cancer, with overall 5-year survival rates of about 8% (American cancer society cancer facts & 024, 2024). This dismal prognosis is due to lack of early detection methods, few effective therapies, and a poor understanding of risk factors for this disease. *KRAS* mutations are present in early human pancreatic intra-epithelial neoplasia (PanIN) lesions as well as invasive PDAC, and these activating mutations are generally accepted as the initiating event in PDAC progression (Kanda et al., 2013). However, PDAC development in mice carrying the *KRAS* ^G120/+^ mutation is slow (Hingorani et al., 2003), suggesting that other genetic or environmental factors are needed for tumor promotion. While established risk factors for PDAC include family history, inherited genetic mutations, chronic pancreatitis, smoking, alcohol use, obesity and diabetes, the role of other factors including non-alcoholic fatty pancreatic disease, viral hepatitis and environmental exposures is becoming clear (Pandol et al., 2012; Wood et al., 2022; Michalak and Malecka-Wojciesko, 2023; Peduzzi et al., 2025).

The rates of PDAC—which currently ranks as the 10^th^ most common cancer in the U.S. — have been rising and death rates associated with this cancer are expected to increase by 2.4-fold by 2030, making it the second-leading cause of cancer death (Rahib et al., 2014). Globally, PDAC is the 12^th^ most common tumor type but ranks as the 7^th^ leading cause of cancer mortality (Ilic and Ilic, 2022). The rise in PDAC rates mirror the increase in obesity in the U.S., where about three-fourths of the population are overweight or obese (Fryar CD and Afful, 2020). In line with that, population studies suggest that obesity and underlying metabolic dysfunction account for up to 50% of all PDACs (Michaud et al., 2001; Bracci, 2012; Eibl et al., 2018), and findings from animal models support this association (Incio et al., 2016). The timing of obesity seems to further modulate the risk of PDAC: Being overweight or obese in early life and young adulthood is more strongly associated with this cancer than obesity later in life (Li et al., 2009; Genkinger et al., 2015; Nogueira et al., 2017; da Cruz et al., 2019). Although this link between obesity and PDAC is strong, the underlying mechanisms behind this association are not fully understood. Preclinical studies also suggest that obesity reduces the effectiveness of chemotherapy (Incio et al., 2016). However, whether obesity increases mortality among PDAC patients remains unclear as data from epidemiological studies are contradictory (Shinoda et al., 2022).

Evidence from both humans and animal studies show that obesity causes intestinal microbial dysbiosis altering the composition, diversity and function of the gut microbiota (Turnbaugh et al., 2006; John and Mullin, 2016). These alterations in the intestinal microbiome can impact the host metabolism and contribute to weight gain and disease progression. In genetically obese mice (*ob/ob*), the gut microbiota is enriched in Gram-positive and depleted Gram-negative bacteria phyla (Turnbaugh et al., 2006). Introduction of the *ob/ob* mice microbiota in germ free mice resulted in increased body fat mass compared to those receiving lean microbiota (Turnbaugh et al., 2006). These obesity-associated alterations in microbiota have been largely recapitulated in humans (Turnbaugh et al., 2006).

Microbial communities can exert biological effects on tissues they colonize as well as distance sites. Consistent with that, the microbiome has been shown to play a role in the development and progression of PDAC and other cancers (Nilsson et al., 2006; Loo et al., 2017; Fan et al., 2018; Pushalkar et al., 2018; Soto-Pantoja et al., 2021; Chen et al., 2024). One study showed that patients harboring oral pathogens like *Porphyromonas gingivalis* are at higher risk of PDAC (Farrell et al., 2012; Fan et al., 2018). Other studies found microbial dysbiosis in the cancerous pancreas itself (Pushalkar et al., 2018) and a link between intratumor microbial diversity and survival in PDAC patients (Riquelme et al., 2019). However, whether body weight impacts this relationship is understudied, though evidence is emerging. One recent study reported that PDAC progression and resistance to chemotherapy in obese mice is mechanistically linked to the gut microbiome (Kesh et al., 2022).

In this study, we aimed to directly study whether obesity (defined by body weight in mice or body mass index [BMI] in humans) shapes the normal and cancerous pancreas microbiome in mice and PDAC in humans. Specifically, we test the primary hypothesis that obesity reprograms pancreatic microbial diversity and composition, using a mouse model and a pilot exploratory human cohort. We also tested a secondary hypothesis that obesity-linked intra-pancreatic microbiome changes are reflected in tumor immune infiltrates and that, in mice, there is a correlation between gut microbial and intra-pancreatic dysbiosis.

Our study shows that obesity is indeed linked to reprogramming of the intra-pancreatic bacterial community composition in both normal or cancerous pancreas of mice and humans. Notably, both the mouse pancreas and human PDAC showed an obesity-associated decrease in the Pseudomonadota phylum. We also found that the presence of cancer by itself reduced microbial diversity in both the mouse pancreas as well the intestinal microbiota, which was further exacerbated by obesity. Finally, we observed that obesity increased circulating inflammatory cytokines and altered the tumor immune infiltrate in humans and mice.

## Methods

### Human samples procurement-exploratory study

#### Human PDAC specimens and serum samples

De-identified, archived formalin-fixed paraffin-embedded (FFPE) tumor specimens and serum samples from PDAC patients were obtained from the Georgetown University Histopathology and Tissue Shared Resource Biorepository. A total of 12 human pancreatic tissue biopsy samples (6 lean and 6 obese patients) were included. Patient characteristics are detailed in **Table 1**. BMI at diagnosis was used as a cut off to stratify patients into lean and obese groups. The experimental design is shown in **Supplementary Figure S1**.

**Table 1:** Anthropometric and clinical characteristics of PDAC patients.

Patients Characterlstics	Lean	Obese
BMI		
Mean	21.8	32.7
Range	20.1 - 24.2	30.4 - 40.6
Gender		
Male	3	3
Female	3	3
Race		
Caucasian	3	3
African- American	3	3
Age (years)		
Mean	68.2	63.6
Range	59.3 - 71.0	56.5 - 82.6
Stage		
IIA	1	0
IIB	5	6
Treatment-naïve		
Yes	6	6
No	0	0

### Animal study

#### Animals, dietary exposures, and breeding

The LSL-Kras^G12D/+^/P48^Cre/+^ (KC) mouse model of PDAC was used in this study (The Jackson laboratories Strains # 008179 [LSL-Kras^G12D/+^] and 023329 [P48^Cre/+^]). Littermates lacking either the P48^Cre^ or LSL-Kras^G12D/+^ allele or both were used as controls and referred to as WT (‘wild type’) from this point on. Obesity and metabolic dysfunction were induced in KC and WT mice by feeding them an Obesity-Inducing-Diet (OID, AIN93G-based diet 57.1% energy from fat) throughout life (indirectly through maternal feeding before weaning and directly after weaning). Another cohort of KC mice and WT littermates were fed a control nutrient balanced diet (CO, AIN93G-based diet containing 17.2% energy from fat). Diets were purchased from Envigo-Teklad and their composition is detailed in **Supplementary Table S1**.

Animal body weight was recorded weekly for up to 16 weeks of age, though a subset of mice with the KC genotype were loss to follow-up due cancer-induced illness. Obese and lean KC mice were used to monitor pancreatic cancer development and for metabolic studies. Both cohorts of mice were also used to harvest pancreatic tissue and fecal samples for microbiome analyses as described in the following sections. The genotype of each animal was determined commercially using genomic DNA extracted from tail clips by Transnetyx, Inc. All animal procedures followed protocol approved by the Georgetown University Animal Care and Use Committee.

The experimental design is shown in **Supplementary Figure S1**.

#### Metabolic parameters

Glucose tolerance test was performed in 8-10week-old mice. Glucose (2.5g/kg body weight) was administered intraperitoneally to fasted animals and tail blood samples was collected at 0, 15, 30, 60 minutes after injection. Blood glucose levels were measured with an ACCU-CHEK Performa portable glucose meter (Roche Diagnostics).

#### Pancreas histopathological evaluation, PanIn lesion scoring and quantification

The KC mouse model rarely develops invasive PDAC (Hingorani et al., 2003). In this model, most animals develop early PDAC lesions or PanIN (pancreatic intra-epithelial neoplasia). Pancreatic tissue sections harvested between 2 and 4 months of age were fixed in neutral buffered 10% formalin, paraffin-embedded and sectioned (5µm). Sections were deparaffinized with xylene, rehydrated through a graded alcohol series and stained with hematoxylin and eosin (H&E). PanINs were classified blindly by our pathologist following previously published guidelines (Basturk et al., 2015; Veite-Schmahl et al., 2017). To quantify PanIn lesions of different stages, 10 random areas/slide were photographed using an Olympus IX-71 Inverted Epifluorescence microscope at 20x magnification and total number of PanIn were counted using the ImageJ software (National Institute of Health, Bethesda, MD, USA).

#### Immunofluorescence

Human or mouse pancreatic tissues were immunostained for CD80-Cy3 (Abcam, ab254579) and CD4-Cy5 (Diagnocine, GB11064) to identify infiltrating macrophage and T cells populations, respectively. Samples were also counterstained with DAPI. Fluorescent signals were visualized and quantified on whole slide scans using CaseViewer software.

#### Circulating cytokines

Assessment of circulating cytokines in serum of human PDAC patients (IL-6, IL-10, IL-17, IL-21, IL-22, IFN-γ and TNF-α) or in plasma of pancreatic cancer bearing mice (IL-6, IL-10, IL-17 and TNF-α) were performed commercially by Quansys Biosciences. Mouse IFN-γ levels were assessed in-house using the ELISA MAX™ Deluxe Set Mouse (Biolegend # 430804).

#### DNA isolation from FFPE pancreatic tissues and tumors

DNA was aseptically extracted from formalin-fixed paraffin-embedded (FFPE) pancreatic cancer tissue specimens from mice (PanINs) and humans (PDAC). Normal pancreas or pancreatic tumor areas were micro-dissected with 25G needle. The tumor area was dissected using the respective H&E-stained section with the tumor area (marked by our pathologist) as a guide. The wax tumor cells were transferred to a 1.5 mL microtube, rinsed with xylene, incubated at room temperature for 5 minutes and centrifuged at 14.000 rpm for 5 minutes. Following xylene wash, samples were rinsed three times with ethanol before being centrifuged 14.000 rpm for 5 minutes. After wash with ethanol, pellet was incubated at room temperature for 5–10 minutes. 180 ul of ATL buffer and 10%proteinase K was added and incubated at 55°C with 500 rpm shaking. To inactivate proteinase K, the samples were heated at 95°C for 10 minutes and before being treated at 37°C for 90 minutes with RNase. The samples were allowed to cool down at room temperature and 1 volume of phenol-chloroform were added, vortexed and centrifuged at 1400 rpm for 5 minutes. The aqueous layer was transferred to a new tube and 0.2 volume of 10 M ammonium acetate, 0.8 volume of glycogen and 2 volume of 100% ethanol were added and incubated at -20°C overnight. The samples were centrifuged at 14.000 rpm for 20 minutes, supernatant removed and pellet was allowed to dry. The pellet was resuspended in 100 ul of RNAse free water and concentration was measured using Nanodrop.

Mouse fecal samples, collected at 10 weeks of age, were placed into a MoBio PowerMag Soil DNA Isolation Bead Plate. DNA was extracted following MoBio’s instructions on a KingFisher robot.

### 16S sequencing

Pancreatic (normal or tumor) and fecal bacterial microbiome 16S sequencing was performed by Microbiome Insights Inc. (Vancouver, British Columbia). Bacterial 16S rRNA genes were PCR-amplified with dual-barcoded primers targeting the V4 region (515F 5’-GTGCCAGCMGCCGCGGTAA-3’, and 806R 5’-GGACTACHVGGGTWTCTAAT-3’), as per the protocol of Kozich et al. (2013). Amplicons were sequenced with an Illumina MiSeq using the 300-bp paired-end kit (v.3). Sequences were denoised, taxonomically classified using Greengenes (v. 13_8) as the reference database, and clustered into 97%-similarity operational taxonomic units (OTUs) with the mothur software package (v. 1.39.5) (Schloss et al., 2009), following the recommended procedure (https://www.mothur.org/wiki/MiSeq_SOP; accessed Nov 2017).

The potential for contamination was addressed by co-sequencing DNA amplified from specimens and from 10 each of template-free controls and extraction kit reagents processed the same way as the specimens. Operational taxonomic units were considered putative contaminants (and were removed) if their mean abundance in controls reached or exceeded 25% of their mean abundance in specimens.

Alpha diversity was estimated with the Shannon index on raw OTU abundance tables after filtering out contaminants. The significance of diversity differences was tested with ANOVA. To estimate beta diversity across samples, we excluded OTUs occurring with a count of less than 3 in at least 10% of the samples and then computed Bray-Curtis indices. We visualized beta diversity, emphasizing differences across samples, using Principal Coordinate Analysis (PCoA) ordination. Variation in community structure was assessed with permutational multivariate analyses of variance (PERMANOVA) with treatment group as the main fixed factor and using 9999 permutations for significance testing. All analyses were conducted in the R environment.

### Statistical analysis

Statistical analyses were performed using GraphPad Prism Software. Differences in mortality rates in lean and obese mice were analyzed using Kaplan-Meier survival curves followed by the log-rank test. Two-way ANOVA was used to analyze number of pre-invasive PDAC lesions (group and grade), body weight and GGT data (group, time – with repeated measures), followed by appropriate *post-hoc* analysis. Differences in circulating cytokines, pancreatic CD4 and CD80 cells were analyzed via multiple t-test with Holm-Sidak correction or individual t-tests. Differences in bacterial taxa were performed using multiple t-test with Holm-Sidak correction or individual t-test. Differences were considered statistically significant at P< 0.05. Unless indicated, *n* corresponds to the number of animals used in each experiment.

## Results

### Obesity enhances PDAC development and mortality rates in the KC mouse model

To induce obesity, male and female WT and KC (LSL-Kras^G12D/+^/P48^Cre/+^) mice were fed an obesity-inducing diet throughout life. Cohorts of WT and KC mice fed a healthy control diet were used as controls (diets composition shown in **Supplementary Table S1**). While mice from both genotypes became obese when fed an obesity-inducing diet (P<0.0001, P<0.0001; **Supplementary Figures S2a–f**), KC mice gained less weight compared to WT mice. This difference is likely attributable to tumor-induced weight loss in KC mice. However, mice in the obese group, regardless of genotype, developed metabolic dysfunction manifested as glucose intolerance (P=0.0317, P=0.0223; **Supplementary Figures S2g–l**).

**Figure 1:**
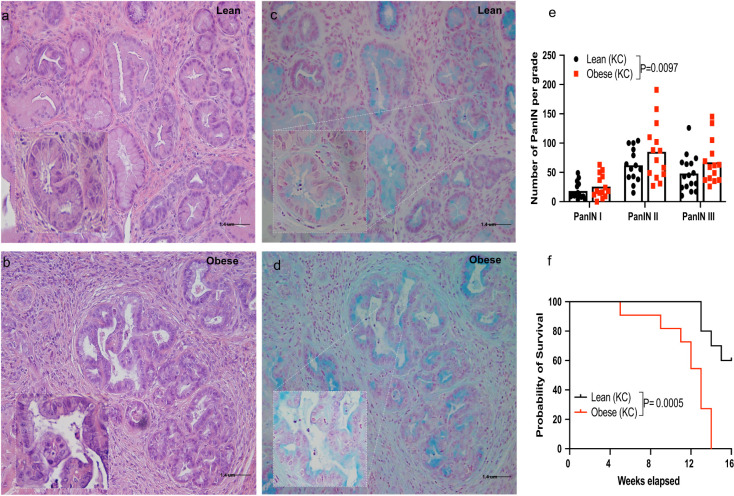
Obesity enhances pancreatic ductal adenocarcinoma (PDAC) development and mortality rates in the KC mouse model. Male and female KC (LSL-Kras^G12D/+^/P48^Cre/+^) mice were fed a control (lean group) or an obesity-inducing diet (obese group) throughout life and monitored for PDAC development up to16 weeks of age (**Supplementary Figure S1**). **(a-d)** Representative histology of pancreatic sections showing PanIN lesions in lean and obese KC mice **(a, b)**, H&E staining; **(c, d)**, Alcian blue staining, 20x [inset 40x] Scale bar 1.4 uM); **(e)** Quantification of PanIN lesions shown by grade and **(f)** survival in lean and obese KC mice (n=14-16/group). Differences in PDAC development between groups were determined by two-way ANOVA (group and PanIN grade). Differences in survival were analyzed using Kaplan-Meier survival curves followed by the log-rank test. Data are shown as mean (top of bar graphs in scatter plots in e) or percentage **(f)**.

Obese KC mice harbored a significantly higher number of pre-invasive PDAC lesions (PanIN, pancreatic intra-epithelial neoplasia) compared to lean KC mice (P=0.0097; **Figures 1a–e**). Survival in obese KC mice was also significantly reduced compared to their lean counterparts (P=0.0005; **Figure 1f**). In line with previous reports (Chang et al., 2017), we found that diet-induced obesity accelerated PDAC progression.

### Obesity modulates the microbial populations in the normal and cancerous pancreas

Recent studies showed that tumors, including PDAC, harbor microbial species that can impact cancer progression (Loo et al., 2017; Riquelme et al., 2019; Arnone et al., 2024; Chen et al., 2024). Because it is well established that obesity modulate the gut microbiome, we examined whether body weight would also impact the microbial populations in the normal or cancerous pancreas, using bacterial 16S rRNA sequencing.

**Figure 2:**
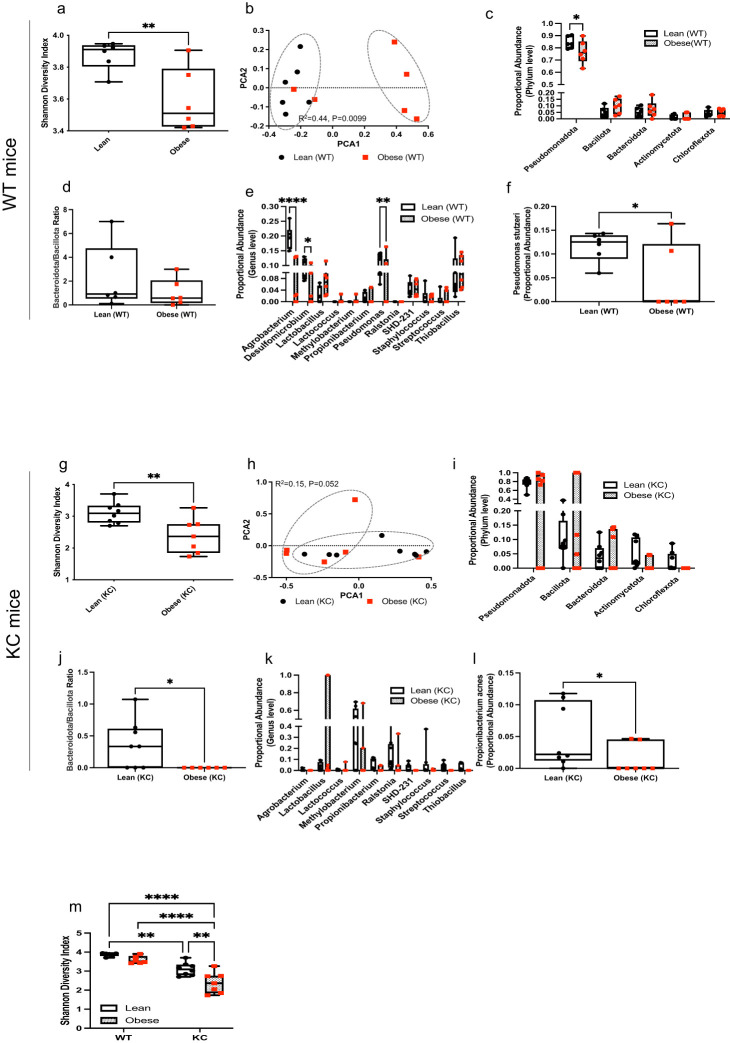
Obesity modulates the microbial composition in the normal and cancerous pancreas in mice. DNA extracted from normal or cancerous pancreatic tissue samples were collected and used for bacterial 16S rRNA profiling. (WT mice): **(a)** Shannon (alpha) diversity index; **(b)** Principal Coordinate Analysis (PCoA) showing community-level (beta) diversity; **(c)** Quantification of proportional abundance of phylum; **(d)** Bacteroidota-to-Bacillota ratio; **(e)** Quantification of proportional abundance of genus; and **(f)** Quantification of bacterial species regulated by obesity in normal pancreas of WT mice (n=6/group). Differences in Shannon diversity was analyzed using t-test. Beta diversity across samples was computed using Bray-Curtis indices and PERMANOVA. Differences in bacterial taxa were performed using multiple t-test with Holm-Sidak correction **(c, e)** or individual t-test **(d, f)**. Data are shown as box plots (min-max and quartiles in **a, c–f**); *P<0.05, **P<0.01, and ****P<0.0001. (KC mice): **(g)** Shannon (alpha) diversity index; **(h)** Principal Coordinate Analysis (PCoA) showing community-level (beta) diversity; **(i)** Quantification of proportional abundance of phylum; **(j)** Bacteroidota-to-Bacillota ratio; **(k)** Quantification of proportional abundance of genus; and **(l)** Quantification of bacterial species regulated by obesity in cancerous pancreas of KC mice (n=7-8/group). Differences in Shannon diversity was analyzed using t-test. Beta diversity across samples was computed using Bray-Curtis indices and PERMANOVA. Differences in bacterial taxa were performed using multiple t-test with Holm-Sidak correction **(i,k)** or individual t-test **(j, l)**. Data are shown as box plots (min-max and quartiles in **g, i-l**); *P<0.05 and **P<0.01. **(m)** Comparison of Shannon (alpha) diversity index (n=6-8/group) between the cancerous (KC) or normal (WT) pancreas. Differences in Shannon diversity was analyzed using two-way ANOVA, followed by Sidak multi-comparison test. Data are shown as box plots (min-max and quartiles in **m**); **P<0.01, and ****P<0.0001.

The normal pancreas of obese males displayed a significant decrease in microbial α-diversity compared to the pancreas of lean mice (**Figure 2a**); P=0.009). There was also significant impact of body weight on community-level composition of bacteria within the normal pancreas (**Figure 2b**); R^2^ = 0.44, P=0.0099). At the phylum level, obese WT mice showed a significant decrease in the proportional abundance of Pseudomonadota compared to the lean group (**Figure 2c**; P=0.0474). The Bacteroidota-to-Bacillota ratio was non-significantly lower in obese mice (**Figure 2d**). Genus-level analysis revealed a decrease in *Agrobacterium* (P**<**0.0001), *Desulfomicrobium* (P=0.0170), *Pseudomonas* (P=0.0055) in the pancreas of obese WT mice compared to lean mice (**Figure 2e**). Notably, obese mice also harbored significantly lower levels of the *Pseudomonas stutzeri* species compared to lean mice (**Figure 2f**; P=0.0268).

Similarly, we observed that the cancerous pancreas of obese KC mice exhibited significantly lower bacterial α-diversity (Shannon index) compared to lean mice (**Figure 2g**; p=0.009). While obesity also impacted community-level composition of bacteria, the interaction between obesity and diversity within the cancerous pancreas was only borderline significant (**Figure 2h**; R^2^ = 0.15, P=0.052). OTUs were aggregated into different taxa and plotted the proportional abundance of the most abundant microbial populations. While no differences at phylum levels were observed (**Figure 2i**), the Bacteroidota-to-Bacillota ratio was significantly reduced in tumors of obese mice (**Figure 2j**; P=0.043). We did not observe differences at genus level taxa between the cancerous pancreas of obese and lean KC mice (**Figure 2k**). However, obese KC mice harbored significantly lower levels of *Propionibacterium acnes* (**Figure 2l**; P=0.0488) compared to lean mice.

We also observed an interaction between obesity and cancer status on the pancreas microbiota. The pancreas of lean WT mice, exhibited the highest bacterial diversity (**Figure 2m**). Compared to this group, the presence of PanIN lesions resulted in a significant decline in microbial diversity independent of body weight (P=0.0032, P<0.0001). Our results also indicate an additive suppressive effect of obesity and cancer on pancreatic microbial diversity (P<0.0001, P=0.0042).

Overall, our results link obesity to modulation of the microbial populations in the healthy and cancerous pancreas in mice.

### Obesity modulates the intestinal microbiome in tumor-bearing and healthy mice

**Figure 3:**
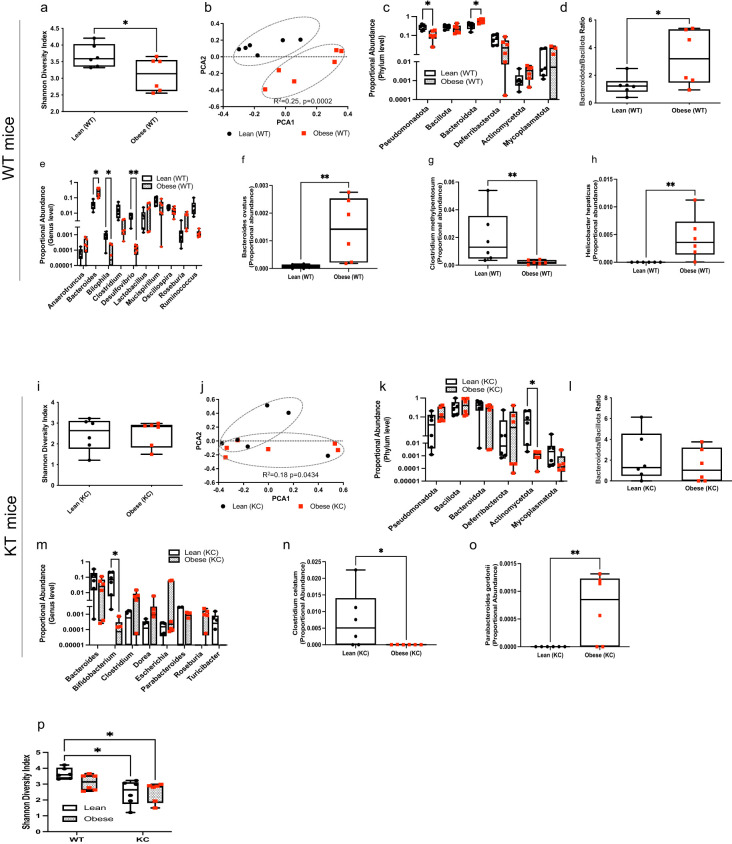
Obesity modulates the gut microbial composition in KC and WT mice. DNA extracted from mouse fecal samples collected at ten weeks of age and used for bacterial 16S rRNA profiling. WT mice: **(a)** Shannon (alpha) diversity index; **(b)** Principal Coordinate Analysis (PCoA) showing community-level (beta) diversity; **(c)** Quantification of proportional abundance of fecal phylum; **(d)** Fecal Bacteroidota-to-Bacillota ratio; **(e)** Quantification of proportional abundance of fecal genus; and **(f-h)** Quantification of fecal bacterial species regulated by obesity in WT mice (n=6/group). Differences in Shannon diversity was analyzed using t-test. Beta diversity across samples was computed using Bray-Curtis indices and PERMANOVA. Differences in bacterial taxa were performed using multiple t-test with Holm-Sidak correction **(c, e)** or individual t-test **(d, f-h)**. Data are shown as box plots (min-max and quartiles in **a, c-h**); *P<0.05 and **P<0.01. KC mice: **(i)** Shannon (alpha) diversity index; **(j)** Principal Coordinate Analysis (PCoA) showing community-level (beta) diversity; **(k)** Quantification of proportional abundance of fecal phylum; **(l)** Fecal Bacteroidota-to-Bacillota ratio; **(m)** Quantification of proportional abundance of fecal genus; and **(n, o)** Quantification of fecal bacterial species regulated by obesity in KC mice (n=6/group). Differences in Shannon diversity was analyzed using t-test. Beta diversity across samples was computed using Bray-Curtis indices and PERMANOVA. Differences in bacterial taxa were performed using multiple t-test with Holm-Sidak correction **(k,m)** or individual t-test **(l, n, o)**. Data are shown as box plots (min-max and quartiles in **i, k-o**); *P<0.05 and **P<0.01. **(p)** Comparison of Shannon (alpha) diversity index (n=6/group) between fecal samples from WT and KC mice. Differences in Shannon diversity was analyzed using two-way ANOVA, followed by Sidak multi-comparison test. Data are shown as box plots (min-max and quartiles in **p**); *P < 0.05.

We examined the impact of obesity on the intestinal microbiome in mice with or without pancreatic cancer. Obese WT mice exhibited a significant decrease in intestinal microbial α-diversity (Shannon index) compared to lean counterparts (**Figure 3a**; P=0.047). Obesity also significantly impacted the gut microbial community composition in WT mice (**Figure 3b**, R^2^ = 0.25, p=0.0002). At the phylum level, lower abundance in Pseudomonadota (P=0.0122) and higher levels of Bacteroidota (P=0.0371) were observed in obese WT mice compared to lean WT mice (**Figure 3c**). Accordingly, the intestinal Bacteroidota-to-Bacillota ratio was significantly higher in WT obese mice as was a genus-level rise in *Bacteroides* (P=0.0469) (**Figure 3d**; P=0.0433). We also observed a decrease in the *Bilophila* (P=0.0431) and *Desulfovibrio* (P=0.0027) genera in obese WT mice compared to lean controls (**Figure 3e**). Consistent with the genus level findings, we detected higher abundance of *Bacteroides ovatus* (P=0.0022). We also observed higher abundance of *Helicobacter hepaticus* (P=0.0022) but lower abundance of *Clostridium methylpentosum* (P=0.0087) species in obese WT mice compared to controls (**Figures 3f–h**).

While obesity did not significantly alter intestinal α-diversity (Shannon index) in KC mice harboring early PDAC lesions (**Figure 3i**), we found a significant impact on community-level composition of the intestinal microbiota (**Figure 3j**; R^2^ = 0.18, P=0.0434) in KC mice. At the phylum level, obese KC mice harbored a lower abundance of Actinomycetota (**Figure 3k**; P=0.0129). Obesity did not alter the Bacteroidota-to-Bacillota ratio in KC mice (**Figure 3l**). Genus-level analysis showed that obesity reduced intestinal *Bifidobacterium* in mice harboring PDAC lesions (**Figure 3m**; P=0.0172). The gut of obese KC mice with pancreatic cancer also harbored significantly lower levels of the *Clostridium celatum* (P=0.0334) but higher levels of the *Parabacteroides gordonii* (P=0.0086) species compared to lean KC mice (**Figures 3n, o**).

Although obesity is a strong driver of reduced microbial diversity in the intestine, the presence of PDAC lesions exerted a further suppressive effect. Lean WT mice, exhibited a significantly higher α-diversity compared to lean (P=0.0109) and obese (P=0.0123) KC mice (**Figure 3p**). This observation suggests that pancreatic cancer by itself is enough to cause a decline in gut microbial richness, independent of body weight.

Our findings show that obesity shapes the intestinal microbiome in tumor-bearing and healthy mice. We also found that presence of both obesity and PDAC lesions interacted to suppress microbial diversity.

### Obesity modulates the intratumor microbiome composition in human PDAC

To confirm findings in the mouse model, we studied the impact of obesity, defined as BMI of 30 or higher, on the PDAC intratumor microbiome in a small cohort of treatment-naïve patients, using 16S rRNA sequencing. Patient characteristics are shown **Table 1**.

**Figure 4:**
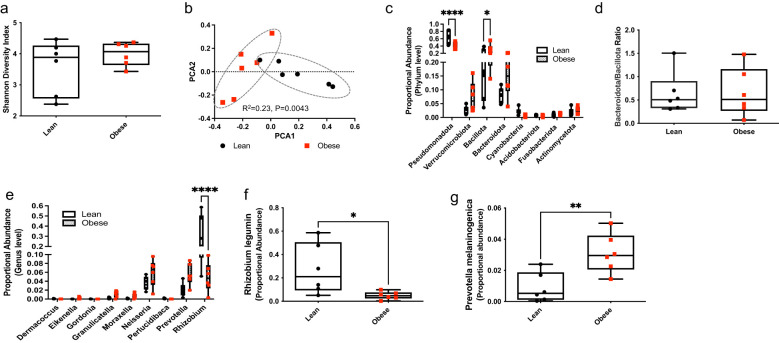
Obesity modulates the intratumor microbiome composition of human PDAC. DNA was extracted from archival treatment-naïve PDAC samples from lean and obese patients and used for bacterial 16S rRNA profiling. **(a)** Shannon (alpha) diversity index; **(b)** Principal Coordinate Analysis (PCoA) showing community-level (beta) diversity; **(c)** Quantification of proportional abundance of phylum; **(d)** Bacteroidota-to-Bacillota ratio; **(e)** Quantification of proportional abundance of genus; and **(f, g)** Quantification of bacterial species regulated by obesity in human PDAC (n=6/group). Differences in Shannon diversity was analyzed using t-test. Beta diversity across samples was computed using Bray-Curtis indices and PERMANOVA. Differences in bacterial taxa were performed using multiple t-test with Holm-Sidak correction **(c,e)** or individual t-test **(d, f, g)**. Data are shown as box plots (min-max and quartiles in **a**, **c-g**); *P<0.05, **P<0.01, and ****P<0.0001.

Although we did not detect an effect of patient BMI at the time of resection on PDAC microbial α-diversity (**Figure 4a**), we found that patient obesity status significantly impacted community-level composition or β-diversity within PDAC tumors (**Figure 4b**, R^2^ = 0.23, P=0.0043). We then aggregated OTUs into different taxa, and plotted the proportional abundance of the most abundant microbial populations. At the phylum level, PDAC from obese patients had significantly lower levels of Pseudomonadota and increased levels of Bacillota compared to lean patients (**Figure 4c**). Despite the increase in Bacillota in PDAC of obese patients, we did not detect differences in Bacteroidota-to-Bacillota ratio between the groups (**Figure 4d**). PDAC from obese patients also displayed reduction in the proportional abundance of the *Rhizobium* genus compared lean patients (**Figure 4e**). Accordingly, we detected a decrease in intratumor levels of *Rhizobium leguminosarum* species in patients with an obese BMI, which also show a significant increase in *Prevotella melaninogenica* species (**Figures 4f, g**). These findings are consistent with our observations in mice and confirm that obesity is linked to changes in bacterial communities within the human cancerous pancreas.

In line with our findings in mice, we show that obesity is linked to the intratumor microbiome composition in human PDAC. **Supplementary Table S2** summarizes the obesity-linked microbial changes in both humans and mice.

### Obesity alters circulating cytokines and the tumor immune infiltrate in humans and mice

**Figure 5:**
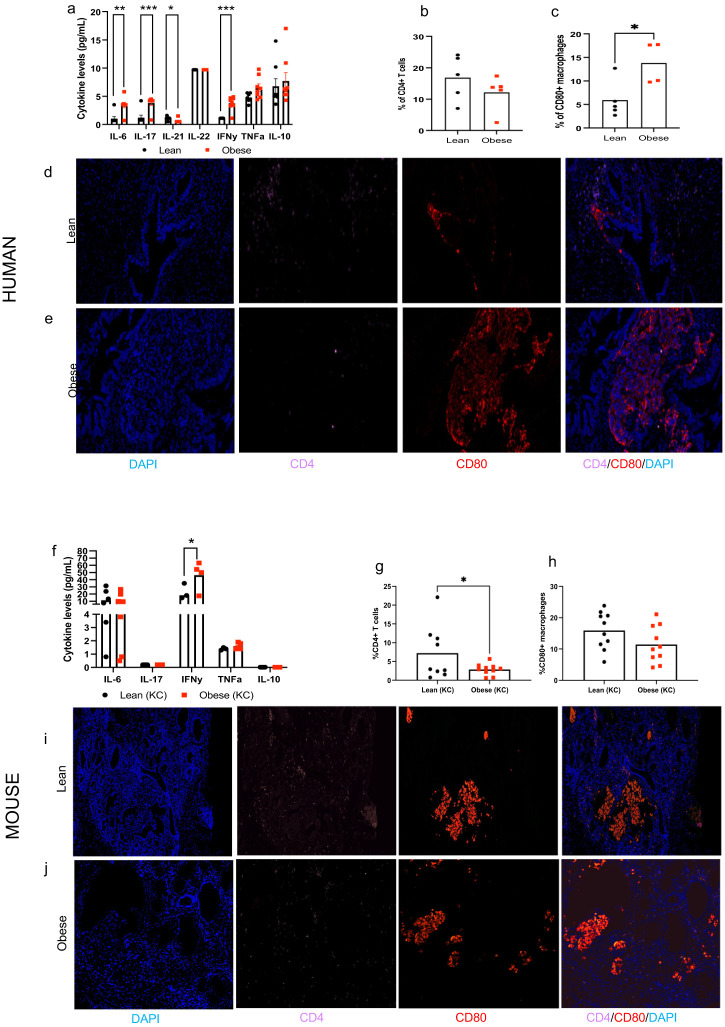
Obesity alters circulating inflammatory cytokines and tumor immune infiltrate in humans and mice. Human: **(a)** Circulating cytokines in lean and obese patients. **(b, c)** Quantification and **(d, e)** representative pictures of the tumor immune cell infiltrate (CD4+ T cells and CD80+ macrophages, 20x) in lean and obese PDAC patients (n=4-5/group). Mouse: **(f)** Circulating cytokines in lean and obese KC mice (n=4-8/group). **(g, h)** Quantification and **(i-j)** representative pictures of the tumor immune cell infiltrate (CD4+ T cells and CD80+ macrophages, 20x) in lean and obese KC mice (n=9-10/group). Differences in cytokine levels were determined multiple t-test with Holm-Sidak correction and immune cell infiltrate was analyzed by individual t-test. Data are show as mean (top of bar graphs in scatter plots) *P<0.05, **P<0.01 and ***P<0.001.

We also determined the impact of obesity on circulating inflammatory cytokines and PDAC immune infiltrate in humans. Obese PDAC patients displayed a distinct inflammatory profile compared to lean patients, with significantly higher levels of circulating pro-inflammatory cytokines, including IL-6, IL-17, and INF-γ (**Figure 5a**, P= 0.0051, 0.0038, 0.0012, respectively). We also observed a non-significant increase in TNF-α levels (P=0.0874). Conversely, levels of the immunoregulatory cytokine IL-21 were significantly lower in obese patients (P=0.0340), with no significant differences observed for IL-22 or IL-10. Furthermore, the composition of immune cells infiltrating the tumors differed between groups. PDAC of obese patients showed a significantly higher percentage of CD80+ macrophages (P=0.0259) and a non-significant decrease in CD4+ T cells compared to PDAC of lean patients (**Figures 5b–e**).

Data in mice largely replicated the effects of obesity on circulating inflammatory cytokines and tumor immune infiltrate in humans. Obese KC mice also displayed significantly higher circulating levels of IFN-γ (P=0.0270) compared to lean KC mice (**Figure 5f**). We also observed higher levels of TNF-α, but results did not reach statistical significance (P=0.11). No significant differences were observed in circulating levels of IL-6 between groups while levels of IL-17 and IL-10 were undetectable. Pre-invasive PDAC lesions from obese KC mice showed significantly lower percentage of infiltrating CD4+ lymphocytes compared to lean mice (P=0.0369), though no significant differences in CD80+ macrophages were observed in contrast to human PDAC (**Figures 5g–j**).

We found that obesity alters circulating cytokines and the intra-pancreatic tumor immune infiltrate in humans and these findings are largely replicated in mice.

## Discussion

Microbial communities exert biological effects directly on tissues they colonize, as well as indirectly in distance sites. It has been reported that PDAC harbors a microbiome that can modify cancer outcomes including tumor progression, response to therapy and overall survival (Pushalkar et al., 2018; Riquelme et al., 2019). Less studied is whether patient characteristics impact this relationship. Although it is well-established that the gut microbiota is modulated by dietary intake and obesity, it is not known whether these factors influence the pancreatic microbiome. Ours is the first study to examine and show the influence of obesity (defined by body weight in mice or BMI in humans) on the microbial diversity in the normal and cancerous pancreas. Overall, we observed that obesity reprograms the bacterial community composition in the normal pancreas and PDAC in mice and in our pilot human cohort. Obese mice and humans each had a distinctive pancreatic microbiome signature with specific bacterial phylum, genus and species. We also noted that the presence of cancer by itself reduced microbial diversity in both the pancreas as well the intestinal microbiota in mice. This reduction in microbial richness was further was exacerbated by obesity. We further observed that obesity increased inflammatory cytokines and altered the tumor immune infiltrate in humans and mice.

Populations studies clearly link obesity to increased incidence of PDAC in humans, though they report conflicting findings on whether obesity increases PDAC mortality (Shinoda et al., 2022), likely due to the high lethality of this cancer. Obesity and associated gut microbiota are also reported to increase PDAC incidence and reduce the effectiveness of chemotherapy (Incio et al., 2016; Kesh et al., 2022) in preclinical mouse models. In line with that, we observed an increase in PDAC incidence and mortality in obese KC mice which was accompanied by microbial dysbiosis in the gut and pancreas.

We detected a decreased in the *Bifidobacterium* genus in the gut of obese KC mice. Similarly, we observed a reduction in bacterial species belonging to the *Clostridium* genus in both WT and KC mice. Microbes within these two genera have anti-cancer properties through their ability to induce apoptosis and inhibit the cell cycle (Sexton et al., 2022). These commensal probiotic species also produce SCFA, metabolites known to improve gut leakiness, decrease inflammation in the pancreas and other digestive organs (Temel et al., 2023). However, whether these and other intra-pancreatic bacterial communities do indeed play a role in PDAC initiation, progression and mortality in obese individuals remains to be determined.

A recent study showed that a high-fat diet associated gut microbiota produces leucine and promote cancer progression by activating polymorphonuclear myeloid-derived suppressor cell production in both breast cancer and melanoma models (Chen et al., 2024). Consistent with that, we observed a marked obesity-induced increase in circulating inflammatory cytokines and alterations in the tumor immune-environment in both in humans and mice. While the relationship between pro-inflammatory cytokines and PDAC is complex, these signaling molecules can promote cancer development, progression, and metastasis. For instance, IL-6, TNF-alpha and IL-17 can activate cancer growth by promoting immune escape via signaling pathways such as MAP/STAT and NF-κB. IFN-γ can also induce immune evasion by increasing PD-L1 expression, which inhibits immune response (Li et al., 2022). However, whether the presence or absence of certain bacterial communities, and the microbiota-derived metabolites, in the gut of obese mice or humans play a functional role in PDAC immune infiltration needs to be fully investigated.

Both human PDAC tissues and the normal mouse pancreas exhibited an obesity-induced decrease in the Pseudomonadota phylum. In our mouse model, most of the microbial communities we detected in either the normal or cancerous pancreas were also present in the intestine and in some instances with the same obesity-driven directional changes in both sites (e.g. Pseudomonadota). These findings suggest a potential translocation of gut bacterial communities that can colonize the pancreas as previously shown (Pushalkar et al., 2018). However, future animal studies using fecal transplantation will need to be conducted to answer this question. Interestingly, *Agrobacterium* and *Rhizobium*, two related genera of bacteria previously shown to be abundant in both the WT and KC mouse pancreas (Pushalkar et al., 2018), were downregulated in the pancreas of both obese mice and humans compared to their lean counterparts. Although our findings are consistent with previous reports showing the presence of *Rhizobium* in the intra-tumor space (Pushalkar et al., 2018) in fresh frozen PDAC, this bacterial species is more commonly found as a legume symbiont. Thus, future studies are needed to demonstrate causality of *Rhizobium* as a driver of PDAC.

Notably, we observed an increase in *Prevotella melaninogenica* in the PDAC tissue of obese human patients. This species is an anaerobic commensal that colonize the oral mucosa in early life. Oral *Prevotella melaninogenica* and other *Prevotella* strains can easy access the gastrointestinal tract via swallowing and are known to play a role in gastrointestinal diseases (Kononen and Gursoy, 2021). A number of studies have also linked *Prevotella melaninogenica* and other members of the *Prevotella* genus to chronic inflammation and promotion of oral squamous carcinoma and tumor of the digestive tract (Kononen et al., 2022). These studies report that *Prevotella* microbes activate IL-17, NF-κB and other inflammatory signaling pathway and are relevant to patient clinicopathological status and cancer progression (Larsen, 2017; Kononen and Gursoy, 2021).

Despite the overall adverse effects of obesity on cancer incidence and progression, some immune therapies are reported to be more efficacious in obese subjects (Vick et al., 2023). Indeed, it was recently reported that impaired immunoediting in obese rodents enhances tumor immunogenicity, making them sensitive to immunotherapy (Piening et al., 2024). In agreement with these findings, obesity is linked with improved progression-free survival and overall survival in male melanoma patients treated with immunotherapy (McQuade et al., 2018). *Prevotella melaninogenica*, which we show is increased in the PDAC tissue of obese patients, has been shown to synergize with and improve immune checkpoint blockade therapies in multiple myeloma (Calcinotto et al., 2018). However, whether obese PDAC patients are more responsive to immunotherapies or whether the *Prevotella melaninogenica* species play a role in PDAC progression and outcomes remains to be determined.

Although our findings are intriguing, there are several limitations in our study. First, the KC mouse model used here does not faithfully recapitulate the PDAC development in humans as invasive PDAC is rarely seen in this model (Hingorani et al., 2003). Thus, comparisons between the microbial alterations in our mouse model and human patients with invasive PDAC require conservative interpretation. Another limitation is the small sample size in our PDAC patient cohort which composed exclusively of stage II, treatment-naïve, tumors and therefore should be considered exploratory. Thus, our microbiome analysis findings will need to be validated prospectively in larger human cohorts (preferably in using freshly resected PDAC specimens) that incorporate confounder adjustment (e.g. age, sex, antibiotic use), which we were unable to perform in this pilot study.

Another limitation is that, while our mouse model of obesity was induced via a high-fat diet feeding, human obesity is likely multi-factorial and more complex than the just the amount of dietary fat consumption. Thus, we cannot completely rule out a confounding effect of dietary intake in obese patients. Furthermore, other lifestyle factors linked with obesity such as lack of exercise and use of certain medications (antibiotics or those used to treat metabolic syndrome) can influence the gut microbiome. However, it currently not known if these factors affect the intra-tumoral or normal pancreatic tissue microbial populations.

It is also important to note that microbiome analysis of low biomass tissues like the pancreas is still challenging and the potential for environmental contamination is a limitation for this type of data. In our study, this was addressed by co-sequencing DNA amplified from specimens and from each of template-free controls and extraction kit reagents processed the same way as the specimens. However, selecting an appropriate contamination threshold to balance sensitivity/specificity tradeoffs for low-biomass environments can be difficult. Although we are confident that our method minimized contaminant reads in our datasets, we cannot completely rule it out. However, if contaminants (e.g. from reagents) did exist, they would present in all samples, and not aggregated by BMI or obesity status, because our specimens were processed in a single batch (one for human and one for mouse samples).

Because our findings are largely correlative, future experiments are needed to investigate whether the obesity-induced pancreatic microbiota plays a functional role on PDAC progression. It also remains to be seen whether interventions to shift the gut or the pancreas-specific microbiome translates into real changes tumorigenesis in PDAC models. For instance, experiments using microbiota transplantation into germ-free PDAC mouse models will be required to establish a causal relationship. In humans, studies using large, multi-center cohorts, that take factors (e.g. dietary intake, exercise and antibiotic use) influencing the microbiome into account are required to confirm our findings and rule out potential confounding in obese PDAC patients. Incorporation of KPC models to model invasive PDAC and use of isocaloric high-fat diets to separate caloric excess effects from adiposity will also strengthen the findings of the current study.

Despite these limitations and remaining questions, our study shows there is a striking link between obesity and bacterial diversity of the normal and cancerous pancreas in both mice and PDAC patients. We also report an interaction between obesity and cancer that modifies the gut microbiome. Although we do not yet know whether obesity-associated microbes play a role in tumor development and progression in PDAC patients, treatments to improve outcomes will likely require consideration of patient characteristics such as BMI and associated life-style factors. Our findings and recent published reports (Riquelme et al., 2019) raise the tantalizing possibility that pancreatic microbiome diversity could be a major component in individualized PDAC therapeutic interventions to help reduce the burden of this disease. Further investigating obesity-driven microbial differences will provide important insights for personalized treatments for PDAC patients.

## Data Availability

The raw data supporting the conclusions of this article will be made available by the authors, without undue reservation.

## Supplementary material

Supplementary Figure 1 Experimental Design: **(A)** Male and female mice with either a WT or KC (LSL-Kras^G12D/+^/P48^Cre/+^) genotype were fed a healthy control (lean group) or an obesity-inducing diet (obese group) throughout life. Body weights were monitored weekly. Glucose tolerance test was performed between 8–10 weeks of age. KC mice were monitored for PDAC development up to16 weeks of age. At the end of this period, fecal matter, pancreatic tissue (normal and cancerous) and blood were harvested. Normal pancreas, tumors and fecal samples were used for microbiome analysis using 16S rRNA profiling. Blood samples were used to measure cytokine levels. PDAC tumors were used to assess the immune infiltrate. **(B)** Treatment-naïve, de-identified, archived formalin-fixed paraffin-embedded (FFPE) tumor specimens and serum samples from PDAC patients were obtained from the Georgetown University Histopathology and Tissue Shared Resource Biorepository. Patients were stratified by BMI into lean and obese categories. PDAC tumors were used for microbiome analysis using 16S rRNA profiling and to assess the immune infiltrate. Serum samples were used to measure cytokine levels.

Supplementary Figure 2 Longitudinal body weight and metabolic function in WT and KC mice consuming a control or obesity-inducing diet. Male and female mice with either a WT or KC (LSL-Kras^G12D/+^/P48^Cre/+^) genotype were fed a control (lean) or obesity-inducing diet (obese) throughout life. **(a-f)** Longitudinal body weight in WT or KC lean and obese mice shown as aggregated data **(a,d)** or segregated by sex (b-c, e-f). Metabolic function in WT or KC lean and obese mice performed between 8–10 weeks of age: **(g-l)** Glucose tolerance test (GTT) shown as aggregated data **(g,i)** or segregated by sex **(h-i, k-l)**. Longitudinal body weights (n=14–16 group) and GTT curves (n=9-12) were analyzed by two-away (time and group) ANOVA separately for each genotype. Data shown as mean±SEM.
