# Supplementary figures and images for: Obesity reprograms the normal pancreas and pancreatic cancer microbiome in mice and humans
Source: Front Microbiomes. 2025 Jul 28;4:1543144. doi: 10.3389/frmbi.2025.1543144 (PMC12993647; doi:10.3389/frmbi.2025.1543144)

## STUDY DESIGN

### A. Mouse

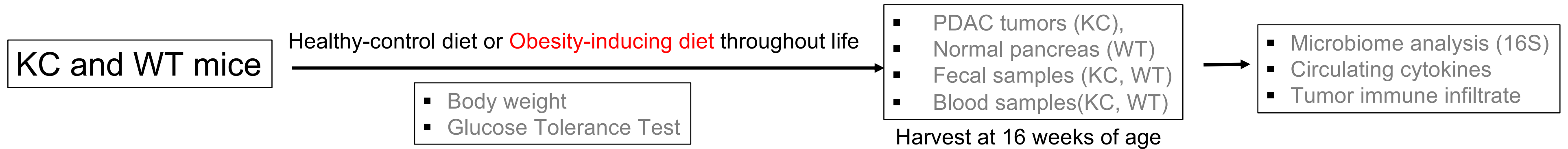

### B. Human

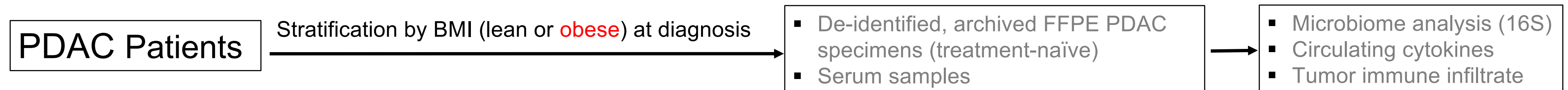

Figure S1

Supplement: Supplementary Figure 1 — Experimental Design: (A) Male and female mice with either a WT or KC (LSL-KrasG12D/+/P48Cre/+) genotype were fed a healthy control (lean group) or an obesity-inducing diet (obese group) throughout life. Body weights were monitored weekly. Glucose tolerance test was performed between 8–10 weeks of age. KC mice were monitored for PDAC development up to16 weeks of age. At the end of this period, fecal matter, pancreatic tissue (normal and cancerous) and blood were harvested. Normal pancreas, tumors and fecal samples were used for microbiome analysis using 16S rRNA profiling. Blood samples were used to measure cytokine levels. PDAC tumors were used to assess the immune infiltrate. (B) Treatment-naïve, de-identified, archived formalin-fixed paraffin-embedded (FFPE) tumor specimens and serum samples from PDAC patients were obtained from the Georgetown University Histopathology and Tissue Shared Resource Biorepository. Patients were stratified by BMI into lean and obese categories. PDAC tumors were used for microbiome analysis using 16S rRNA profiling and to assess the immune infiltrate. Serum samples were used to measure cytokine levels. [file Image1.pdf]

Longitudinal Body Weight

KC

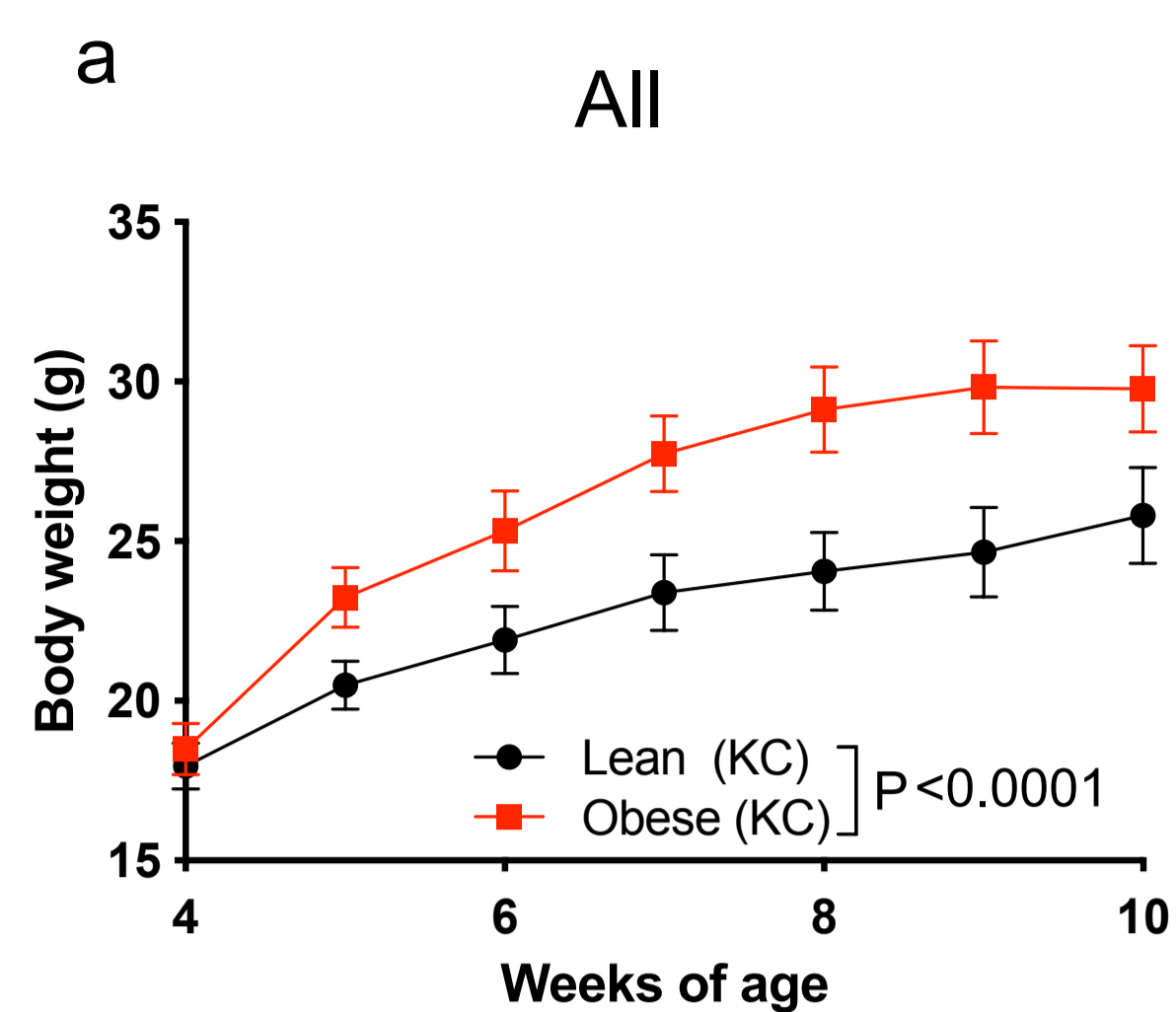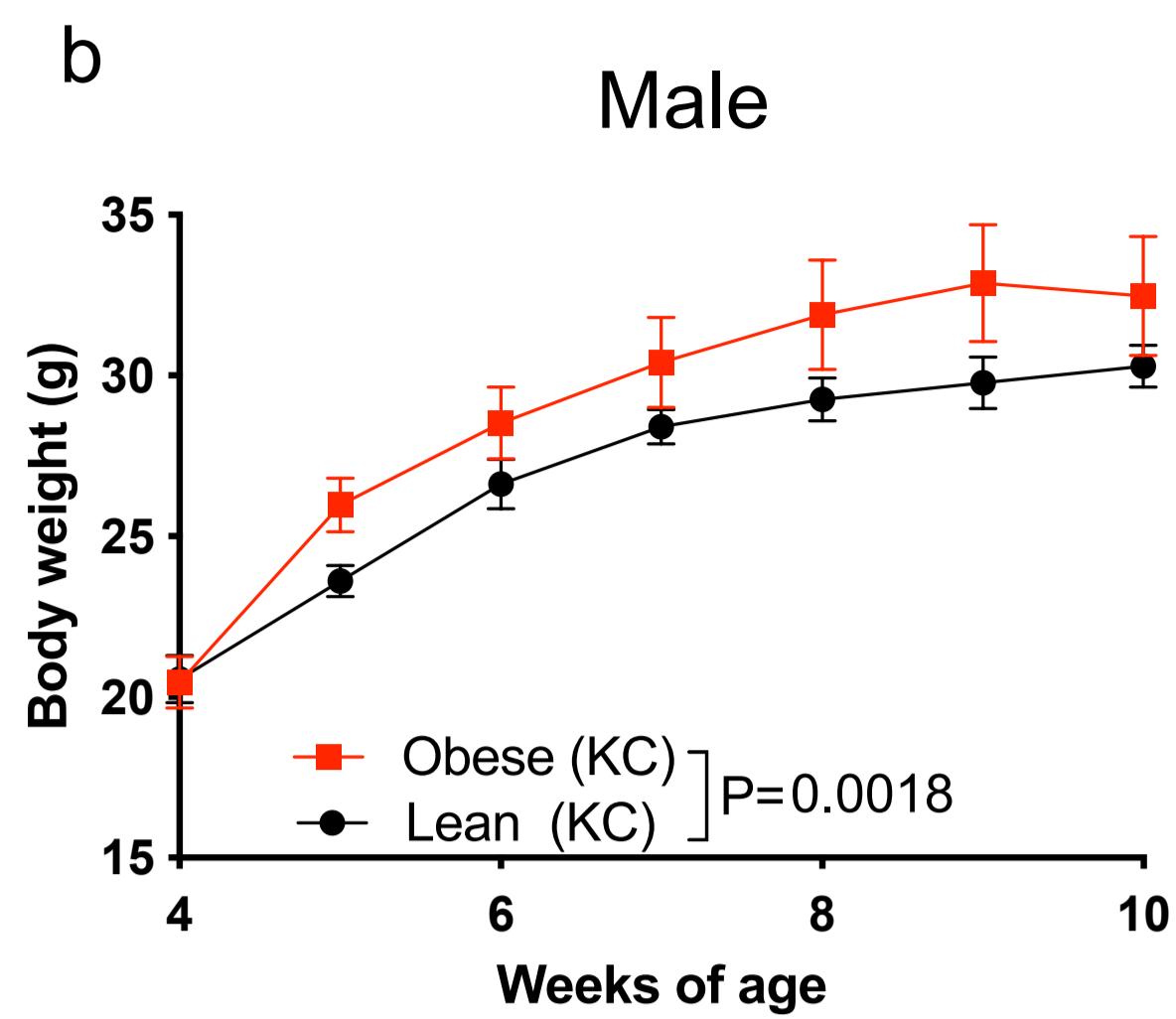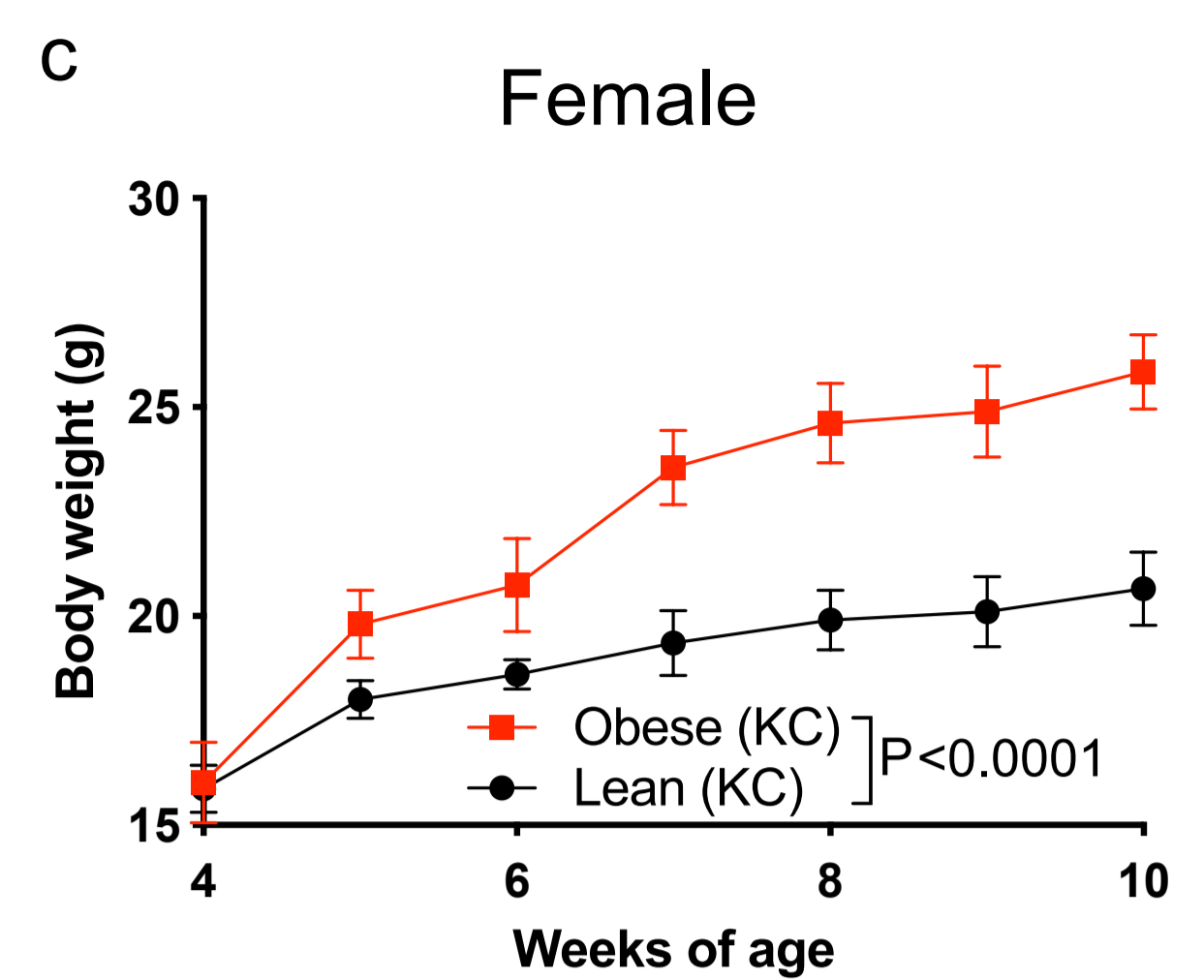

WT

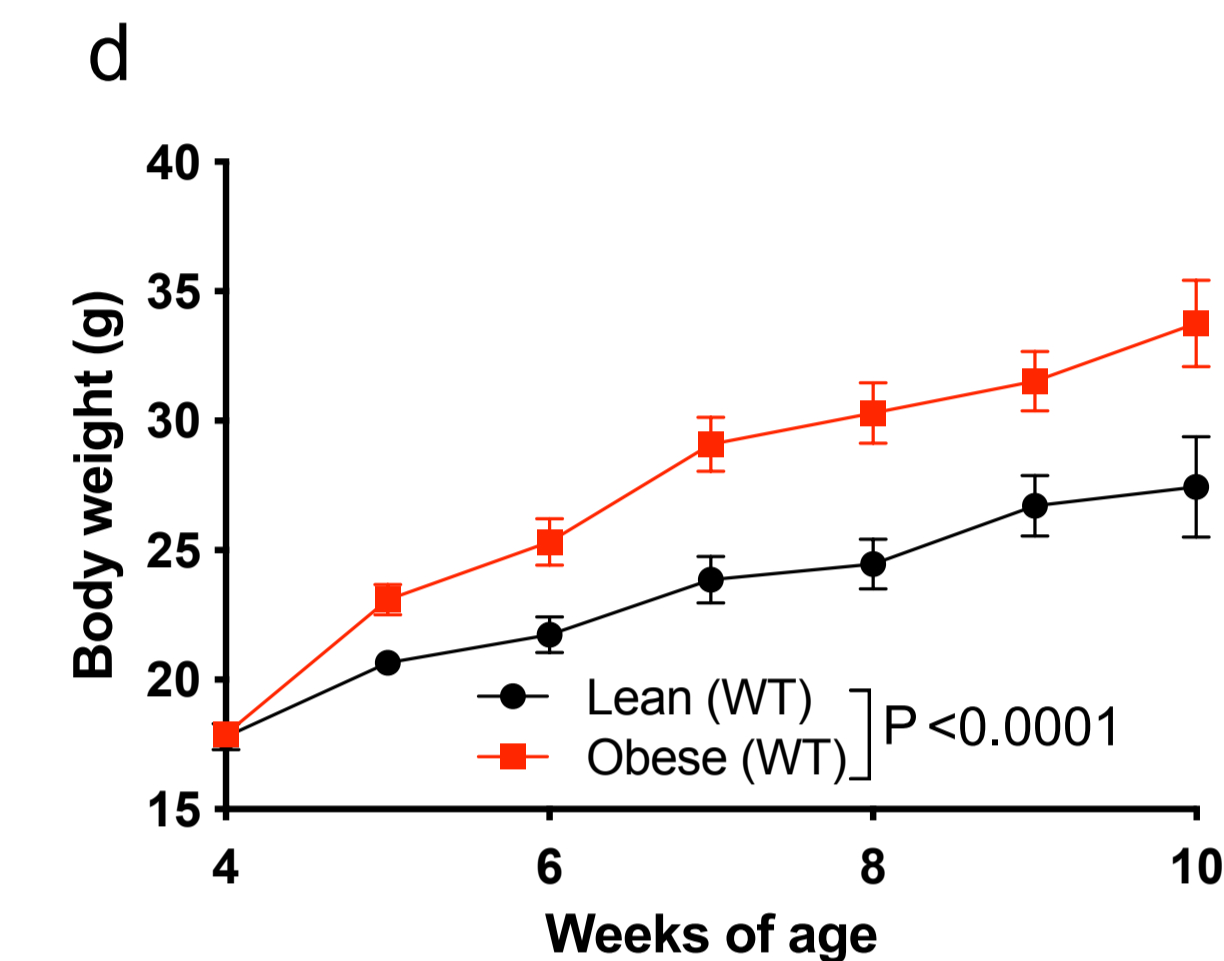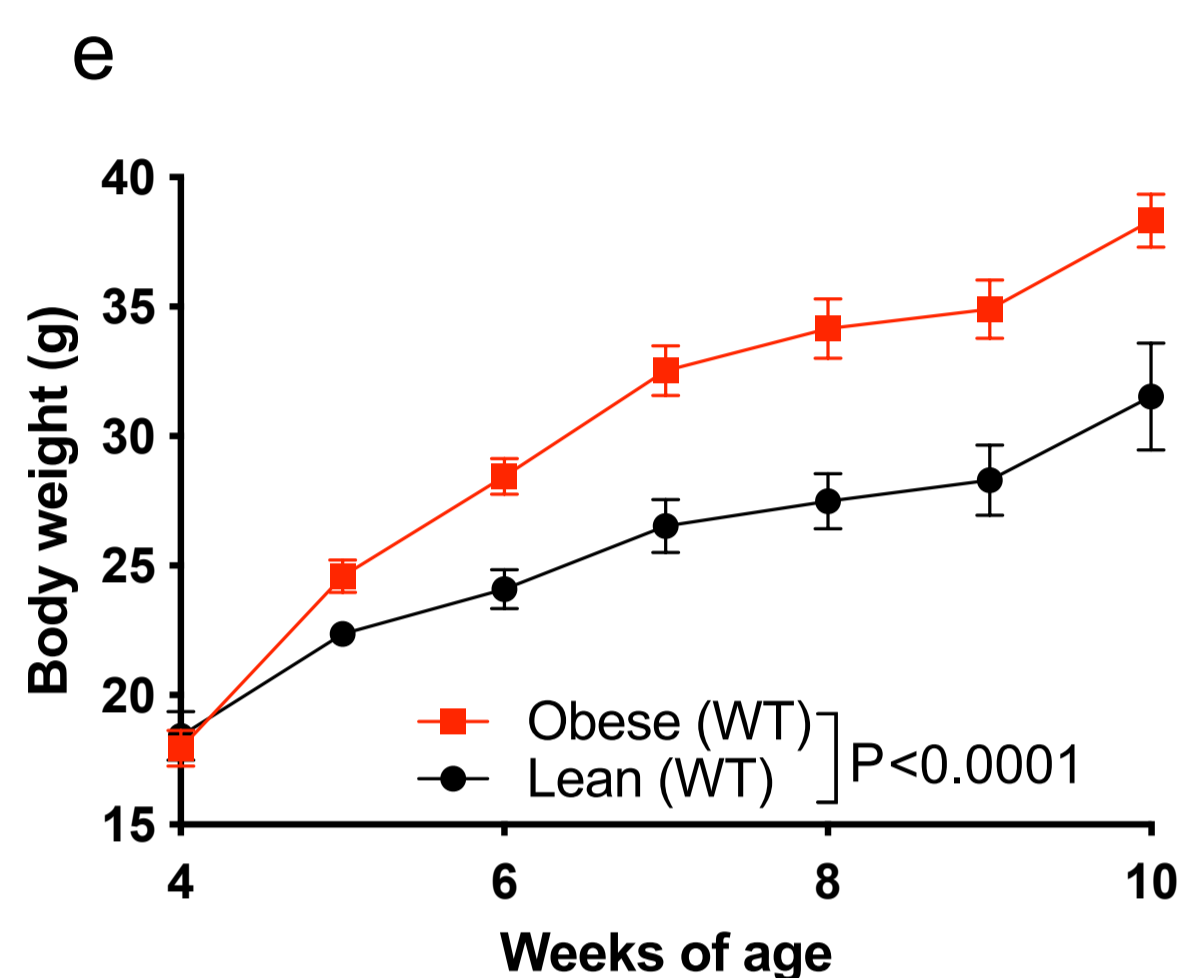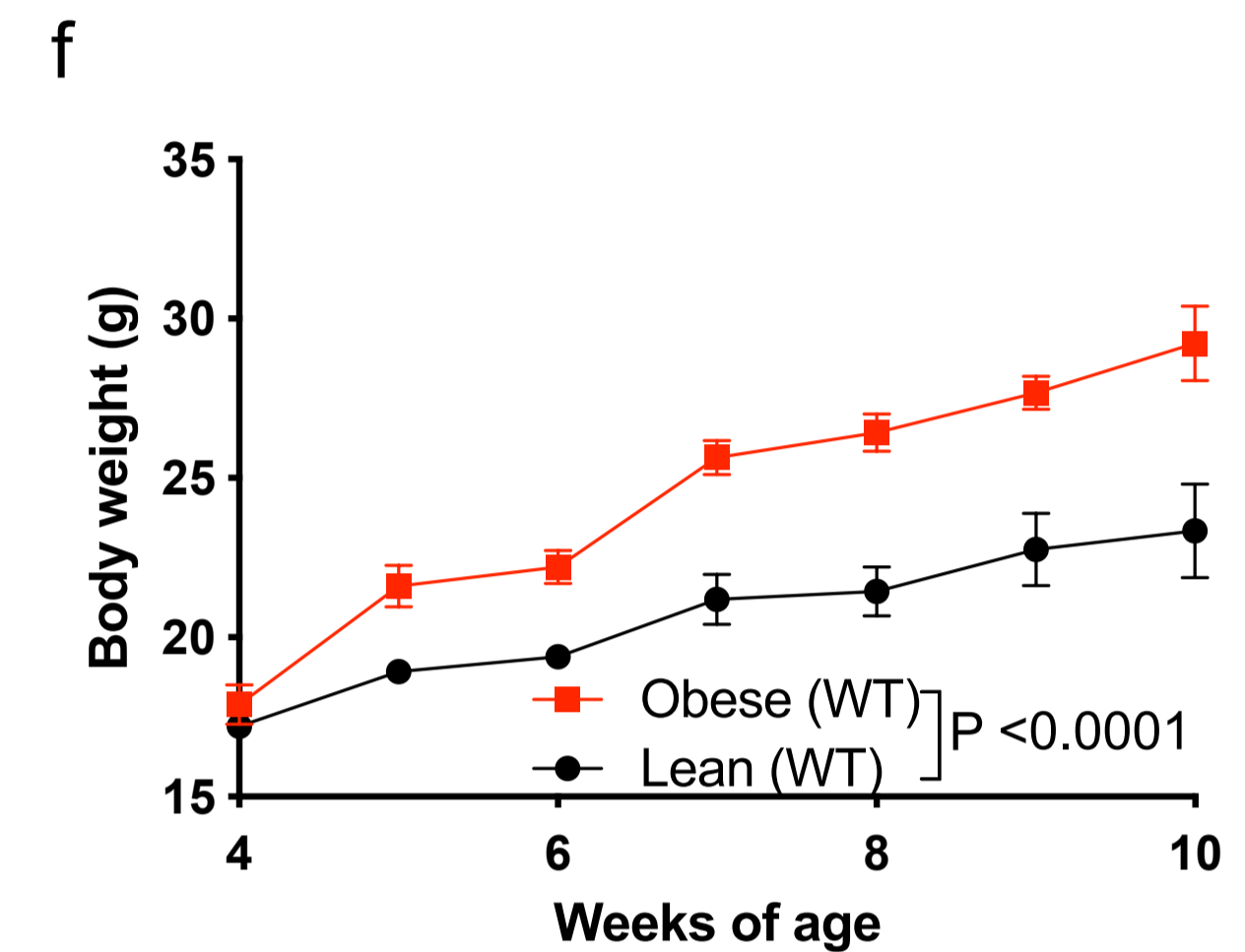

Glucose Tolerance Test

KC

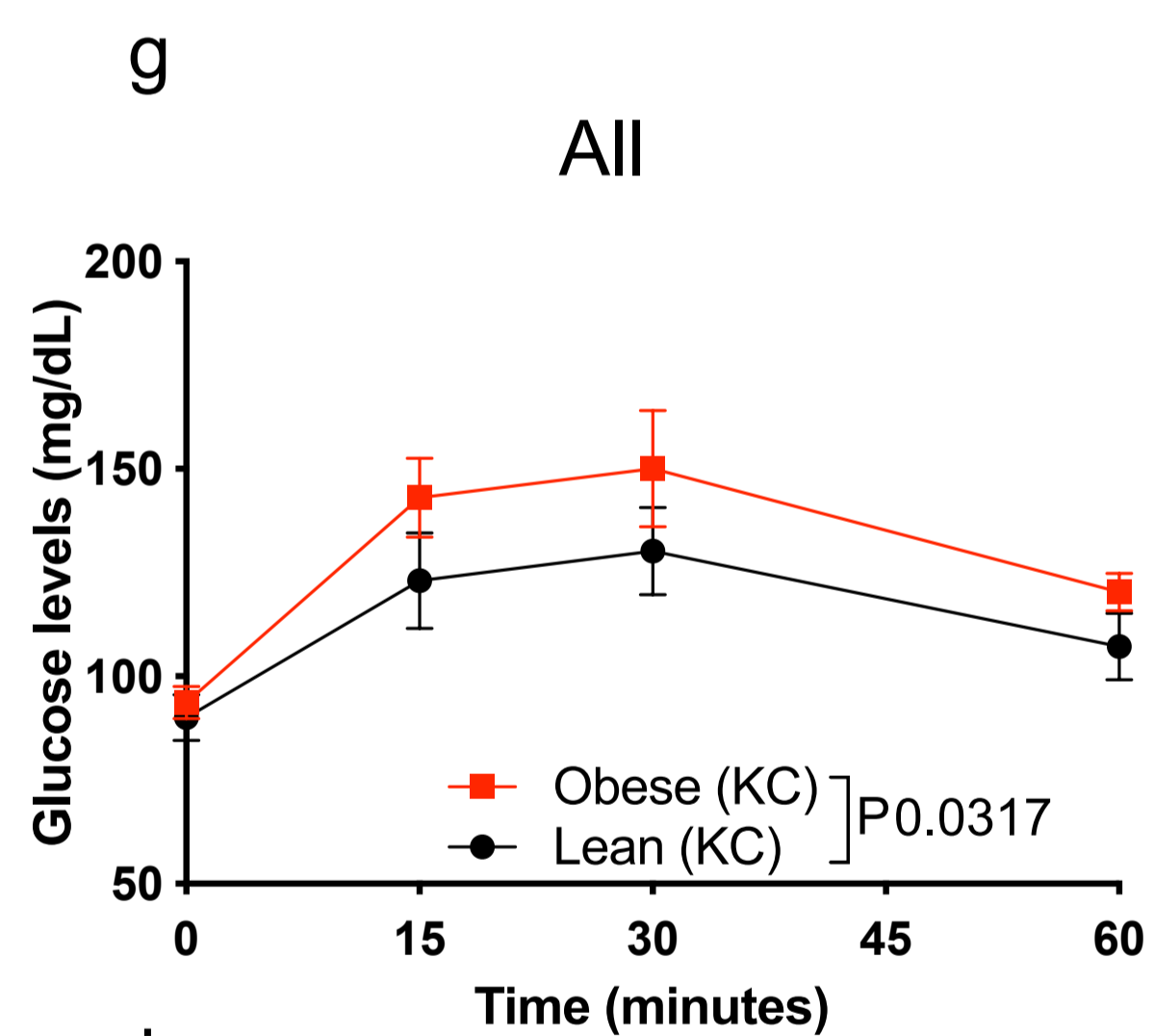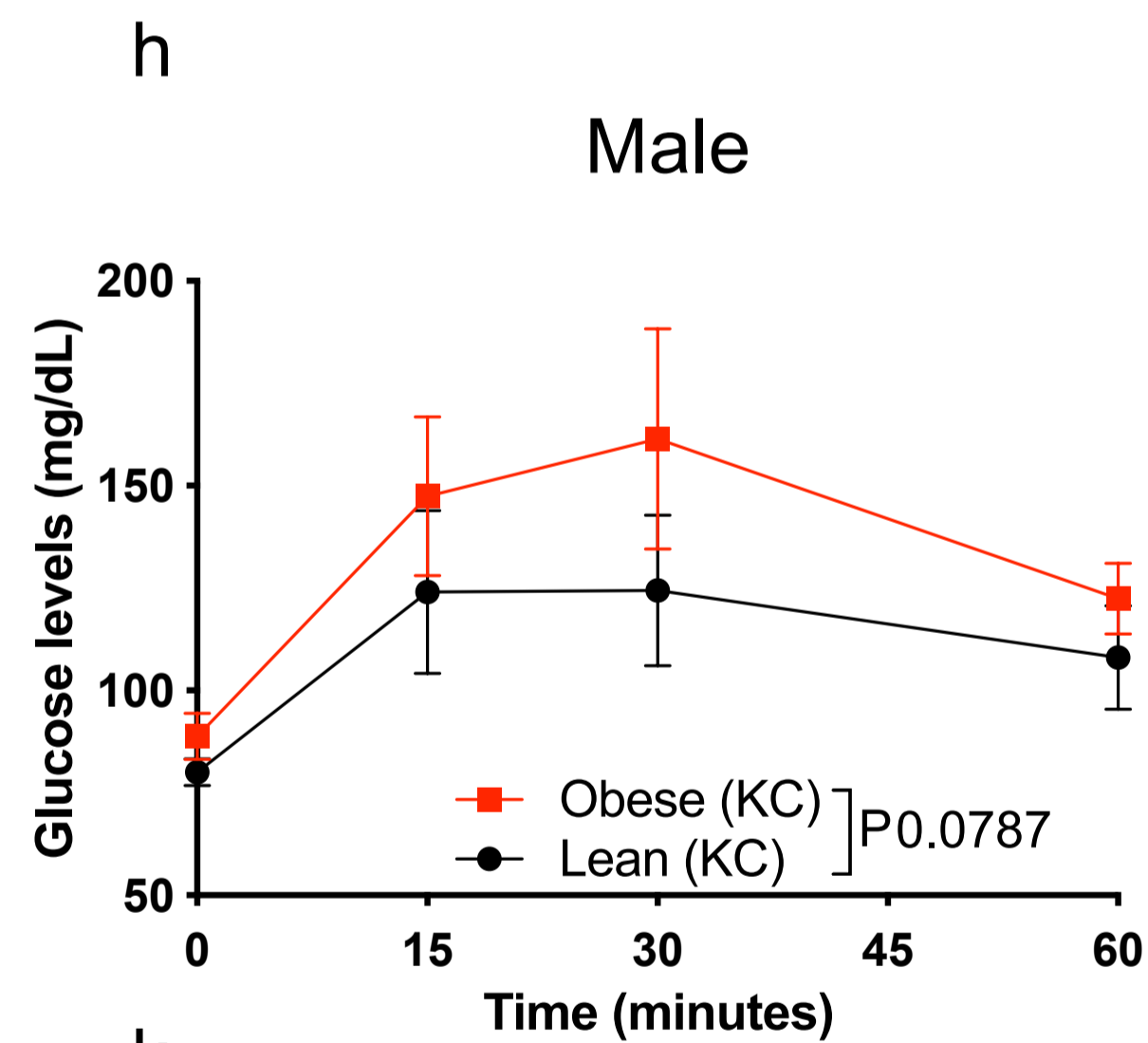

Supplement: Supplementary Figure 2 — Longitudinal body weight and metabolic function in WT and KC mice consuming a control or obesity-inducing diet. Male and female mice with either a WT or KC (LSL-KrasG12D/+/P48Cre/+) genotype were fed a control (lean) or obesity-inducing diet (obese) throughout life. (a-f) Longitudinal body weight in WT or KC lean and obese mice shown as aggregated data (a,d) or segregated by sex (b-c, e-f). Metabolic function in WT or KC lean and obese mice performed between 8–10 weeks of age: (g-l) Glucose tolerance test (GTT) shown as aggregated data (g,i) or segregated by sex (h-i, k-l). Longitudinal body weights (n=14–16 group) and GTT curves (n=9-12) were analyzed by two-away (time and group) ANOVA separately for each genotype. Data shown as mean±SEM. [file Image2.pdf]
