# Supplementary material for: Obesity reprograms the normal pancreas and pancreatic cancer microbiome in mice and humans
Source: Front Microbiomes. 2025 Jul 28;4:1543144. doi: 10.3389/frmbi.2025.1543144 (PMC12993647; doi:10.3389/frmbi.2025.1543144)
Supplement: Supplementary file 3 [file Image3.pdf]

**Ingredients**	g/kg	
	**Control Diet (CO)** (TD.160018)	**Obesity-Inducing Diet (OID)** (TD.160019)
Casein	200.0	288.0
L-Cystine	3.0	2.0
Corn Starch	397.386	-
Maltodextrin	132.0	150.45
Sucrose	100.0	142.0
Corn Oil	50.0	50.0
Lard	20.0	280.0
Cellulose	50.0	20.0
Mineral Mix, AIN-93-MX (94046)	35.0	49.7
Vitamin Mix, AIN-93-VX (94047)	10.0	14.2
Choline Bitartrate	2.50	3.55
TBHQ, antioxidant	0.014	-
Food Coloring	0.1	0.1
% protein by weight (% kcal)	17.7 (18.8)	25.3 (19.3)
% carbohydrate by weight (% kcal)	60.1 (63.9)	31.0 (23.6)
% fat by weight (% kcal)	7.2 (17.2)	33.3 (57.1)
Kcal/g	3.8	5.2

Table S1-Diet Composition
