# Supplementary material for: Obesity reprograms the normal pancreas and pancreatic cancer microbiome in mice and humans
Source: Front Microbiomes. 2025 Jul 28;4:1543144. doi: 10.3389/frmbi.2025.1543144 (PMC12993647; doi:10.3389/frmbi.2025.1543144)
Supplement: Supplementary file 4 [file Image4.pdf]

Taxa	Human PDAC	Mouse pancreas/PDAC	Mouse fecal matter
**Phylum level**			
Pseudomonadota	Decreased	Decreased	Decreased
Bacillota	Increased	No change	No change
Bacteroidota	No change	No change	Increased
Actinomycetota	No change	No change	Decreased
**Genus level**			
Rhizobium	Increased	No change	No change
Agrobacterium	No change	Decreased	No change
Desulfomicrobium	No change	Decreased	No change
Pseudomonas	No change	Decreased	No change
Bacteroides	No change	No change	Increased
Bilophila	No change	No change	Decreased
Desulfovibrio	No change	No change	Decreased
Bifidobacterium	No change	No change	Decreased
**Species level**			
Prevotella melaninogenica	Increased	No change	No change
Rhizobium leguminosarum	Decreased	No change	No change
Pseudomonas stutzeri	No change	Decreased	No change
Propionibacterium acnes	No change	Decreased	No change
Bacteroides ovatus	No change	No change	Increased
Helicobacter hepaticus	No change	No change	Increased
Clostridium methylpentosum	No change	No change	Decreased
Clostridium celatum	No change	No change	Decreased
Parabacteroides gordonii	No change	No change	Increased

Table S2. Summary of obesity-linked microbiome changes in humans and mice
